# Whole genome survey of coding SNPs reveals a reproducible pathway determinant of Parkinson disease

**DOI:** 10.1002/humu.20840

**Published:** 2008-10-13

**Authors:** Balaji S Srinivasan, Jaleh Doostzadeh, Farnaz Absalan, Sharareh Mohandessi, Roxana Jalili, Saharnaz Bigdeli, Justin Wang, Jaydev Mahadevan, Caroline LG Lee, Ronald W Davis, J William Langston, Mostafa Ronaghi

**Affiliations:** 1 Department of Statistics, Stanford UniversityStanford, California; 2 Department of Computer Science, Stanford UniversityStanford, California; 3 Stanford Genome Technology Center, Stanford UniversityPalo Alto, California; 4 The Parkinson's InstituteSunnyvale, California; 5 Department of Molecular Biology and Genetics, Cornell UniversityIthaca, New York; 6 Department of Biochemistry, Yong Loo School of Medicine, National University of SingaporeSingapore

**Keywords:** whole genome association, Parkinson's disease, PD, Parkinson, pathway association, axon guidance, candidate pathways

## Abstract

It is quickly becoming apparent that situating human variation in a pathway context is crucial to understanding its phenotypic significance. Toward this end, we have developed a general method for finding pathways associated with traits that control for pathway size. We have applied this method to a new whole genome survey of coding SNP variation in 187 patients afflicted with Parkinson disease (PD) and 187 controls. We show that our dataset provides an independent replication of the axon guidance association recently reported by Lesnick et al. [*PLoS Genet* 2007;3:e98], and also indicates that variation in the ubiquitin-mediated proteolysis and T-cell receptor signaling pathways may predict PD susceptibility. Given this result, it is reasonable to hypothesize that pathway associations are more replicable than individual SNP associations in whole genome association studies. However, this hypothesis is complicated by a detailed comparison of our dataset to the second recent PD association study by Fung et al. [*Lancet Neurol* 2006;5:911–916]. Surprisingly, we find that the axon guidance pathway does not rank at the very top of the Fung dataset after controlling for pathway size. More generally, in comparing the studies, we find that SNP frequencies replicate well despite technologically different assays, but that both SNP and pathway associations are globally uncorrelated across studies. We thus have a situation in which an association between axon guidance pathway variation and PD has been found in 2 out of 3 studies. We conclude by relating this seeming inconsistency to the molecular heterogeneity of PD, and suggest future analyses that may resolve such discrepancies.

## Introduction

Many of the recent genome-wide association (GWA) studies of complex traits have failed to find individual SNPs of reproducibly large effect. An increasingly popular hypothesis for this phenomenon is that variation at the pathway level is more consistently correlated with variation in a trait than variation in individual SNPs. To investigate this hypothesis, we developed a novel method for ranking pathways by their degree of association with a complex trait. We applied this method to a new whole genome association study of Parkinson disease (PD) to provide an independent replication of the previously reported association between axon guidance and PD. However, we also show that this pathway association is statistically significant in only 2 out of 3 studies, complicating the hypothesis of pathway reproducibility. Idiopathic PD is likely to result from a combination of environmental and genetic factors. With regard to the latter, a great deal of research has focused on finding functionally significant polymorphisms that increase the probability of developing PD, with both candidate gene [Grevle et al., 2000; Le and Appel, 2004; Marx et al., 2003; Mellick et al., 1999; Nicholl et al., 1999; Xu et al., 2002; Zheng et al., 2003] and GWA studies [Maraganore et al., 2005] conducted in recent years. While the candidate genes were identified on the basis of involvement in a biological pathway plausibly linked to the degenerative process that underlies PD, the GWA studies were designed to be unbiased scans for associated SNPs across the genome.

However, problems have arisen with both of these approaches. While several candidate genes like *LRRK2* (MIM# 609007), *SNCA* (MIM# 163890), *DJ1* (HUGO-approved symbol *PARK7*; MIM# 602533), *PINK1* (MIM# 608309), and Parkin (HUGO-approved symbol *PARK2*; MIM# 602544) have been implicated in one or more varieties of familial Parkinsonism [Klein and Schlossmacher, 2007], no single genetic susceptibility factor has yet been shown to consistently increase the risk for PD. Similar inconsistencies have been observed with the recent GWA studies of PD, in that separate investigators were unable to replicate the SNP associations reported in either the Fung et al. [2006] or the Maraganore et al. [2005] studies ([Elbaz et al., 2006; Perez-Tur 2006] and [Clarimon et al., 2006; Goris et al., 2006; Li et al., 2006; Myers, 2006], respectively). It is useful to put the problems of the candidate gene and GWA approaches in perspective by regarding them as two extremes on a continuum. The candidate gene approach specifies “hard priors” that constrain the search space to a small subset of all possible genetic predictors. Conversely, the GWA approach discards all prior information, opening itself up to the problem of massively multiple testing. It is likely that a better solution lies in an intermediate between these two extremes, in which gene function is used to flexibly constrain the set of possible genotype-to-phenotype maps. An important step in this direction was recently taken by Lesnick et al. [2007], who performed a “candidate pathway” analysis to demonstrate that variation in the axon guidance pathway (KEGG:04360; see Kyoto Encyclopedia of Genes and Genomes; www.genome.jp/kegg) could predict PDrelated traits. Specifically, they found that fitting different kinds of regression models to a matrix of SNP dosage variation in the axon guidance pathway allowed prediction of PD susceptibility, survival times, and age-of-onset. However, because they did not have a systematic means of comparing the association strengths of different pathways, Lesnick et al. [2007] could not show that axon guidance variation was significantly more associated with PD than variation in other pathways.

Here, we address this issue by providing an algorithm for ranking individual pathways by their degree of association with PD susceptibility. We show that it is possible to control this ranking for the critical confound of pathway size, thereby preventing pathways from being deemed “strongly associated” simply because many of their SNPs were assayed on a chip. We apply our techniques to both existing WGA studies [Fung et al., 2006; Maraganore et al., 2005] of PD, as well as a new genome wide screen for coding SNPs associated with idiopathic PD, in which we used the Affymetrix MegAllele Genotyping Human 20 K cSNP chips (Affymetrix, Santa Clara, CA) to interrogate approximately 20,000 SNPs (70% of which were coding) in 187 cases and 187 controls. This chip is particularly suitable for a pathway-based analysis as it contains more coding (and hence genic) SNPs than mapping chips in which most features are in nongenic regions [Jorgenson and Witte, 2006], such as the Affymetrix 500K (Affymetrix). Finally, we present a detailed comparative analysis of all three datasets to determine which SNPs and pathways show reproducible associations.

## Materials and Methods

Our GWA study was performed with 374 Caucasians between 50 and 70 years of age, consisting of 187 controls and 187 cases with PD. Institutional Review Board (IRB) approval was obtained and a consent form was signed by all patients participating in this study. Patients were diagnosed with PD if they satisfied the following seven criteria: 1) at least 3 out of the 4 following cardinal features of PD on neurological examination (resting tremor, rigidity, bradykinesia, and postural instability); 2) a clear response to levodopa; 3) an absence of clinical features suggesting atypical parkinsonism; 4) age of onset over 50 years; 5) a disease duration of 10 years or less (to avoid survivor bias); 6) non-Hispanic white; and 7) no familial history of PD. Control subjects were non- Hispanic whites without a history of known neurodegenerative disorder. Each control was matched to a case of the same age and gender.

### Genotyping

Genomic DNA was extracted from blood and quantified by spectrophotometer (Qiagen, Hilden, Germany). Extracted DNA has a 260/280 spectroscopic absorbance ratio between 1.7 and 2, and the DNA quality was also evaluated using gel electrophoresis. Genotyping was performed with the Affymetrix MegAllele Genotyping Human 20 K cSNP chip (Affymetrix), and genotype calls were performed with the Targeted Genotyping Analysis (GTGS) package (Affymetrix). The 20 K cSNP chip uses the previously described molecular inversion probe technology [Absalan and Ronaghi, 2007; Hardenbol et al., 2003] to interrogate approximately 20,000 nonsynonymous cSNPs across the human genome. By selecting a focused content panel of coding SNPs, we concentrated our attention on the class of SNPs that were most a priori likely to be biologically important [Jorgenson and Witte, 2006].

To genotype the cSNPs, an Affymetrix MegAllele Genotyping Human 20 K cSNP was used (Affymetrix), which utilizes a single molecular probe per SNP locus, designed to incorporate two regions of DNA that are complementary to the genomic DNA on either side of the SNP locus. The probes can be used in a single panel for hybridization to genomic DNA. When the molecular inversion probe has hybridized to the target SNP, it forms a circular DNA molecule with a single-base gap. The SNP is located at this single-base gap. The reaction is split into four aliquots and a different dNTP is added to each aliquot. The single-base gap is filled by a polymerization/ligation reaction. Different dNTPs are, therefore, used to provide allele differentiation between the aliquots. Exonuclease digestion of the extraneous linear DNA results in the selection of only circularized DNA to participate further in the assay. These probes are then, in turn, linearized and released from the genomic DNA. The linearized probe has undergone an inversion reaction and it can now serve as template for subsequent amplification reactions. Each probe also contains a unique tag sequence that is complementary to a sequence on a GeneChip microarray. Following PCR, the amplified products are hybridized to the Affymetrix GeneChip Universal Tag Array under a single set of conditions. The unique advantage conferred by this technology is the ability of the covalently closed circular probe to suppress the cross-reactivity of competing hybridizations. This is achieved during the exonuclease enzymatic step that digests all linear DNA, leaving only circularized probes unaffected. It is at this stage that all other incomplete probe sequences and genomic DNA are eliminated from taking any further part in the assay without affecting the signal from the circular DNA and ultimately SNP sequence detection. The microarrays were scanned by the Affymetrix GeneChip Scanner.

Data from the Affymetrix chips were stored in the Laboratory Information Management Systems (LIMS) system. The tracking database linked each sample ID, each probe pool ID, each plate barcode tracking information, and each chip ID used for detection of every sample and probe pool. Tag information from the probe pool ID was used to extract signal feature data from the .cel files for each of the A,C,G,T channels for every marker genotyped on each sample. The tracking database stored information relating to the quality control (QC) metric of the chip hybridization and washing process, including border feature variation, chip to chip variation, and variation among standard controls distributed within each chip. This information was linked to the genotype call as a flag to pull up any processing-related issues. In addition, metrics relating to each chip, such as background level, noise on the background level, and normalization information was recorded and linked to the raw genotype signal data. A cluster base-caller has been used which uses the four signal measurements for each genotype and the allele information for the marker to perform cluster analysis based on an expectation-maximization (E-M) algorithm. The resulting calls have confidence scores that can be mapped to the accuracy of the call using reference sequencing on a small number of markers in a few samples. In addition, the ability to monitor all four bases for each genotype produced a signal-tonoise (S/N) metric as well for every call, which is a further measure of the integrity of the call. This S/N metric comprised a built-in quality control for the entire genotyping process for every single call made. Finally, the cluster analysis algorithm incorporated information relating to the chip-processing quality metrics to flag any quality control problems in that process.

### Genotyping Validation With Pyrosequencing

Pyrosequencing primers for SNP analyses were designed by SOP3, a free web-based software (http://imgen.ccbb.pitt.edu/sop3/). Single-strand DNA preparation and sequencing primer hybridization were performed semiautomatically using a Vacuum Prep Tool and Vacuum Prep Worktable (Biotage AB, Uppsala, Sweden) as described before [Gharizadeh et al., 2006]. Pyrosequencing [Ronaghi et al., 1998] was performed on an automated plate-based benchtop pyrosequencing (PSQ) system (Uppsala, Sweden) at a dispensing pressure of 625 mbar with 4-msec open time and 65-sec cycle time. The nucleotide dispensation order was set for each SNP. The sequencing primers and the pyrosequencing SNP dispensation orders can be found online (Table A: Amplification and Sequencing Primers; www-sequence.stanford.edu:16080/pyrosequencing). The sequence results were obtained in pyrogram formats.

### Data Cleaning

SNP genotyping yielded an initial 374 by 19,628 matrix, where rows represent individuals, columns represent coding SNPs, and entries represent SNP genotypes. After removing monomorphic SNPs and rows/columns containing a high fraction of no-calls for genotype, we were left with a 360 by 14,111 dimensional matrix *X* in which 14 subjects (10 controls and four cases) were dropped. This curated matrix had a genotyping efficiency (fraction of nonmissing values) of 97.3%, and was compared to the corresponding Boolean response vector *Y*, which had 177 controls and 183 cases coded as 0 and 1, respectively.

### SNP-Level Analysis

We began by conducting a univariate analysis to find individual SNPs correlated with the case/control label. Target alleles for SNPs from different studies were obtained from dbSNP build 127 [Wheeler et al., 2007] and lexicographically ordered to facilitate comparison of allele frequencies. That is, in each case the X allele was alphabetically first while the Yallele was alphabetically second, with the primer sequence used to resolve inconsistencies among C/G and A/T polymorphisms. We next tested each SNP for deviation from Hardy-Weinberg equilibrium (HWE) [Salanti et al., 2005] using Fisher's exact test as implemented in the genetics package [Warnes et al., 2007] in R, and checked the validity of the missing-completely-at-random (MCAR) assumption by calculating the Fisher test P value for the 2 × 2 table of case/control responses vs. missing/nonmissing data. We then computed several measures of association between each SNP and the case/control response vector: the (natural) log odds ratio [Agresti, 2002] and associated 95% confidence interval (CI), Fisher's exact tests for genotypic (FG) and allelic association (FA), and logistic regression for dominant (D), additive (A), and recessive (R) dosage models. SNPs that had log odds ratio CIs wider than three were omitted as these corresponded to contingency tables with low counts in one or more of the cells. Following Lesnick et al. [2007], the best (minimum) P value over these five association models was retained and SNPs were ranked by this P value. We termed this minimum P value the “main effect” of the SNP on the trait and the corresponding model the “best model” of the SNP (Table 1). The full version of Tables 1–4 are available as Supplementary Material.

**Table 1:** SNPs With the Highest Individual Associations With PD*

rsID	Gene a	Position	Function	Log OR (95% CI)	Best P value (model)	X/Y alleles	X case frequency	X control frequency
CV1604913^b^	-	2q21.3	-	0.84 (0.53–1.15)	7.0668e-08 (FA)	C/T	0.48	0.29
rs5952606	LOC392451	Xp11.3	Similar to ribosomal protein L19	1.23 (0.75–1.71)	2.4418e-07 (FA)	A/G	0.2	0.07
rs12505221	LOC152586	4q31.1	Similar to RIKEN cDNA 4933434I20	−0.84 (−1.18 to −0.5)	9.9462e-07 (FA)	A/T	0.64	0.8
rs12577167	ENSG00000187918	11p15.4	-	−0.77 (−1.2 to −0.35)	7.4294e-06 (FG)	A/G	0.78	0.88
rs7611703	-	3p26.3	-	0.69 (0.36–1.02)	2.1147e-05 (D)	C/T	0.38	0.23
rs736118	STRA6 (FLJ12541, PP14296)	15q24.1	Stimulated by retinoic acid gene 6 homolog (mouse)	−1 (−1.47 to −0.52)	2.7152e-05 (FA)	C/T	0.82	0.92
rs7591849	WDSUB1 (WDSAM1, FLJ36175, UBOX6)	2q24.2	WD repeat, sterile alpha motif and U-box domain containing 1	0.64 (0.34–0.95)	3.4855e-05 (FA)	G/T	0.49	0.34
rs1799999	PPP1R3A	7q31.1	Protein phosphatase 1, regulatory (inhibitor) subunit 3A (glycogen and sarcoplasmic reticulum binding subunit, skeletal muscle)	−0.9 (−1.33 to −0.47)	3.9379e-05 (FA)	G/T	0.79	0.9
rs9455190	LOC442230	6q13	-	1.02 (0.49–1.55)	4.1803e-05 (FG)	C/T	0.94	0.86
rs7229727	LOC390823	18p11.3	-	−0.62 (−0.93 to −0.32)	6.2968e-05 (FA)	A/G	0.54	0.69
rs6022903	BCAS1	20q13.2–q13.3	Breast carcinoma amplified sequence 1	−1.55 (−2.36 to −0.73)	6.7128e-05 (FA)	C/T	0.92	0.98
rs963075	SERPINB10	18q21.3	Serpin peptidase inhibitor, clade B (ovalbumin), member 10	−0.68 (−1.01 to −0.34)	8.1699e-05 (FA)	A/G	0.21	0.34

* Rank order is determined by the P-value of the best model fit (see text in Table 1). Full tab-delimited tables with SNP-level associations for our study as well as the Fung et al. [2006] and Maraganore et al. [2005] studies are available as Supplementary Material online.

a Some genes indicated with the GeneCards LOC identifier (www.genecards.org/index.shtml) or the Ensembl identifier (www.ensembl.org/Homo_sapiens/index.html). Alias gene symbols shown in parentheses.

b rsID not available.

**Table 4:** SNPs From the Most Highly Ranked Pathways in Our Study*

Pathway	rsID	Gene	Position	Function	Log OR (95% CI)	P value	X/Y alleles	X case frequency	X control frequency
Axon guidance	rs3770208	EPHA4	2q36.1	EPH receptor A4	0.5 (0.8–0.2)	0.0002268 (D)	A/G	0.489	0.367
Axon guidance	rs9867325	ENSG00000-181882	3q22.3	-	0.61 (0.95–0.26)	8.65e-05 (D)	C/G	0.308	0.195
Axon guidance	rs2278107	EPHA7	6q16.1	EPH receptor A7	1.26 (2.1–0.42)	0.0019968 (FG)	C/T	0.066	0.0196
Axon guidance	rs3801583	-	7q21.11	-	−0.79 (−0.14 to −1.44)	0.017328 (A)	C/T	0.918	0.961
Axon guidance	rs3862618	ROBO3	11q24.2	Roundabout, axon guidance receptor, homolog 3 (Drosophila)	−0.37 (0.05 to −0.79)	0.040451 (R)	C/T	0.831	0.874
Axon guidance	rs17288108	NTN4	12q22–q23	netrin 4	0.35 (0.75 to −0.06)	0.032642 (R)	A/G	0.863	0.817
Histidine biosynthesis	rs7297245	HAL	12q22–q24.1	Histidine ammonia-lyase	0.05 (0.42 to −0.32)	0.044123 (R)	C/T	0.212	0.204
Histidine biosynthesis	rs2073440	HECA (HDC,HDCL, HHDC, dJ225E12.1)	6q23–q24	Headcase homolog (Drosophila)	−1.09 (−0.1 to −2.08)	0.033752 (FG)	A/C	0.960	0.986
Histidine biosynthesis	rs11151964	CNDP1	18q22.3	Carnosine dipeptidase 1 (metallopeptidase M20 family)	−0.61 (−0.18 to −1.05)	0.0045739 (A)	A/G	0.102	0.174
T-cell receptor signaling	rs785467	PIK3R3	1p34.1	Phosphoinositide-3-kinase, regulatory subunit 3 (p55, gamma)	−0.37 (−0.04 to −0.69)	0.010981 (R)	A/T	0.661	0.738
T-cell receptor signaling	rs9867325	ENSG00000-181882	3q22.3	-	0.61 (0.95–0.26)	8.6564e-05 (D)	C/G	0.307	0.195
Ubiquitin mediated proteolysis	rs1129038	-	15q13	-	1.00 (1.33–0.67)	1.0614e-09 (FG)	C/T	0.477	0.251
Ubiquitin mediated proteolysis	rs1912403	NEDD4	15q21.3	Neural precursor cell expressed, developmentally downregulated 4	−0.67 (−0.15 to −1.19)	0.0040213 (FG)	C/T	0.065	0.120

* Variation in these SNPs drives the overall association at the pathway level. Note that most of these SNPs have nominal P values, which place them rather far from the top of the univariate SNP ranking in Table 1, underlining the importance of considering SNPs in a multivariate, pathway context. Some genes are indicated with the Ensembl identifier (www.ensembl.org/Homo_sapiens/index.html). Alias gene symbols are shown in parentheses.

### Pathway-Level Analysis

Many whole genome association studies conclude by noting that strongly associated SNPs fall within a given pathway. Rather than invoking this information a posteriori, it is useful to bring it in at the beginning of the analysis. Thus motivated, we developed a statistical test for ranking pathways by their degree of association, which controls for the confound of pathway size.

### Regressing Labels on Dosages

**Figure 1:**
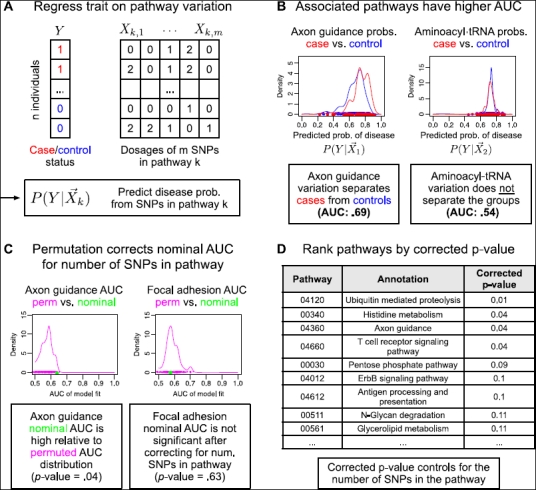
Identifying pathway determinants of disease. **A:** For each pathway, we extracted the SNPs that mapped to genes in that pathway and formed a dosage matrix in which columns were coded according to the best model of the corresponding SNP (see text). We then fit a regression model (see text) to predict the case/control label *Y* from the matrix of SNP dosages *X_k_* of pathway *k*. **B:** The plots contain density estimates of the predicted probability of PD given variation in the axon guidance (KEGG 04360) and aminoacyl-tRNA biosynthesis (KEGG 00970) pathways. Points along the abscissa represent patients, with cases in red and controls in blue. Intuitively, pathways in which variation is strongly correlated with case/control status should show visible separation of the blue and red curves. By measuring this separation with the AUC [Sing et al., 2005], a nonparametric measure of curve separation, we obtained a nominal measure of the fit of the regression model. **C:** An important confound is that pathways with many SNPs will have spuriously high AUCs, a manifestation of the traditional tradeoff between model fit and model complexity [Gentle et al., 2004]. To control for this, for each pathway we generated 500 random dosage matrices with the same number of statistically independent SNPs (see text). We then calculated the AUC of the regression model fit on these 500 random matrices. By estimating the empirical percentile of the nominal AUC relative to this permutation distribution, we computed a corrected P value for the significance of the pathway/trait association. For example, in the figure we show density estimates of the permutation distribution of AUCs in magenta relative to the nominal AUC in green for the axon guidance and focal adhesion pathways. In the left panel, the nominal AUC (green point) for the axon guidance pathway is in the right tail of the magenta permutation distribution. Thus the axon guidance pathway shows a stronger association with PD than a random pathway of the same size. By contrast, the nominal AUC of the focal adhesion is in the middle range of its permutation distribution, indicating that the focal adhesion association is not statistically significant. **D:** The empirical percentile of the nominal AUC relative to the permutation distribution gives us a P-value that measures pathway association, corrected for the number of SNPs in the pathway. This allows us to rank pathways by their degree of association.

We began by mapping SNPs to genes (using ASN flat files from dbSNP [Wheeler et al., 2007] build 127) and genes to KEGG [Kanehisa et al., 2008] pathways. For each pathway *k*, we constructed a predictor matrix *X_k_* of SNP dosages and regressed case/control coding *Y* on SNP dosages (Fig. 1A). We retained only SNPs with minimum P-values less than 0.05 and coded SNPs according to their best association model. For example, SNPs that had their best P-value under the dominant model (D) were coded as (*XX,XY,YY*) → (0,1,1), and similarly for the additive (A) and recessive (R) models; SNPs that had their best P-value under the Fisher allelic or genotypic models were coded as (0,1,2). We used boosted decision trees [Ridgeway, 2007] to perform the regression to allow for scattered missing values in the dosage matrices. Unlike logistic regression, decision trees can naturally handle the occurrence of nonmonotonicity of case proportions as a function of allelic dosage, which is useful for regressing on SNPs that had their best P values under the nonadditive FA or FG models. We calculated the quality of the overall regression of *Y* on the pathway matrix *X_k_* by 10-fold bootstrap cross-validation. Specifically, we randomly assigned 10% of the rows to a test set and 90% into a training set and determined the median area under the receiver operating characteristic (ROC) [Sing et al., 2005] curve (AUC) over 10 such splits. This median AUC *a_k_* was a nominal measure of the association between variation in pathway *k* and the binary trait *Y*, and ranged from 0.5 (no association) to 1.0 (perfect association).

### Equivalent Odds Ratios Allow Comparison of SNP- and Pathway-Level Associations

It is useful to relate the AUC to the odds ratio, which is the standard figure of merit in a GWA study. To do this, let *t* be the true positive rate, *f* be the false positive rate, *R* be the odds ratio, and *A* be the AUC. From the definitions [Agresti 2002; Sing et al., 2005] of these quantities, for a given *t*∈[0,1] and *f*∈[0,1] we obtain:


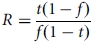
(1)

If instead we regard *R* as fixed, we can consider the set of all *t* vs. *f* values that satisfy this equation to trace out an ROC curve [Pepe et al., 2004]. Though in practice a real ROC curve will have different odds ratios along the curve, the AUC of this “constant odds ratio” ROC curve is useful for comparing the odds ratio (which measures the association of a binary predictor, such as allele presence) with the AUC (which measures the association of a continuous predictor, such as the predicted proportion of cases). To calculate the AUC of the “constant odds ratio” ROC curve we regard the true positive rate *t* as a function of the false positive rate *f* and the odds ratio *R*. We can then calculate the AUC *A* as a function of the odds ratio *R* by integration:


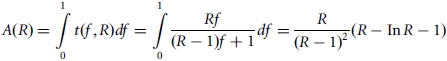
(2)

It can be shown that lim_R→1_ *A*(*R*)50.5 and lim_R→∞_ *A*(*R*) = 1, and that *A*(*R*) is monotonically increasing for R∈[1,∞]. This monotonic relationship indicates that we can quote an “equivalent” AUC for a given odds ratio or vice versa. Tables 2 and 3 contain these equivalent odds ratios for the top ranked pathways in both studies, along with 95% CIs. These values are computed by empirically calculating the inverse mapping from AUCs to odds ratios, *R*(*A*), and then evaluating this mapping at the median AUC and the 0.025 and 0.975 percentile AUCs. The utility of the equivalent odds ratios is that we can directly compare nominal pathway associations to SNP associations in terms of their overall odds ratios rather than quoting the odds ratio at a particular position on the ROC curve.

**Table 2:** Pathways With the Highest Associations With PD in Our Study*

KEGG	Annotation	Nominal AUC (95% CI)	Equivalent log OR 95% CI)	P value
04120	Ubiquitin-mediated proteolysis	0.66 (0.62–0.70)	0.99 (0.74–1.24)	0.01
00340	Histidine metabolism	0.61 (0.57–0.63)	0.68 (0.43–0.82)	0.04
04360	Axon guidance	0.69 (0.65–0.74)	1.18 (0.92–1.54)	0.04
04660	T cell receptor signaling pathway	0.63 (0.58–0.67)	0.81 (0.49–1.02)	0.04
00030	Pentose phosphate pathway	0.62 (0.57–0.65)	0.72 (0.43–0.91)	0.09
04012	ErbB signaling pathway	0.63 (0.61–0.71)	0.82 (0.63–1.32)	0.1
04612	Antigen processing and presentation	0.62 (0.56–0.65)	0.71 (0.36–0.94)	0.1
00511	N-Glycan degradation	0.63 (0.56–0.66)	0.81 (0.34–1.01)	0.11
00561	Glycerolipid metabolism	0.65 (0.61–0.69)	0.90 (0.69–1.18)	0.11
04610	Complement and coagulation cascades	0.69 (0.65–0.74)	1.17 (0.92–1.52)	0.11

**Figure 2:**
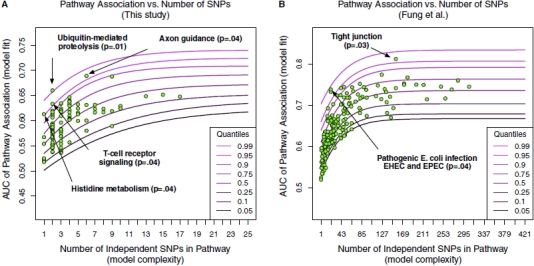
Pathways with more SNPs require higher association levels to reach significance. In each panel, the green points represent pathways and the curves depict the conditional quantiles of the permutation distribution of AUC values given the number of SNPs in a pathway (see text). This plot allows us to determine which (if any) pathways exceed statistical significance in a given study. For example, pathways that lie above the 0.99 quantile curve have a higher nominal AUC than 99% of random pathways with the same number of independent SNPs, and hence have an empirical P value of 0.01. As noted in the text, there are four pathways that exceed the P=0.05 level in our study (**A**), and two that exceed this level in the Fung et al. [2006] study (**B**).

* Pathways are ranked by their P value corrected for the number of SNPs in the pathway; full tables are available as Supplementary Material online. The table contains the nominal AUC and the equivalent nominal log odds ratios along with 95% CIs computed as described in the text in Table 2. This nominal AUC is used to compute the corrected p-value from the AUC permutation distribution as in Figures 1C and 2. Note that the log odds ratios for pathway-level associations are generally higher than those for single SNPs; this is both expected and desirable, as pathway-level associations are based on several SNPs.

KEGG, Kyoto Encyclopedia of Genes and Genomes (www.genome.jp/kegg).

**Table 3:** Pathways With the Highest Associations With PD in the Fung et al. [2006] Study, Ranked by P-Value Corrected for the Number of SNPs in the Pathway*

KEGG	Annotation	Nominal AUC 95% CI)	Equivalent log OR (95% CI)	P value
05131	Pathogenic *E. coli* infection; EPEC	0.74 (0.69–0.76)	1.53 (1.19–1.71)	0.02
04530	Tight junction	0.81 (0.77–0.85)	2.15 (1.82–2.53)	0.03
05130	Pathogenic *E. coli* infection; EHEC	0.73 (0.71–0.76)	1.49 (1.3–1.7)	0.03
00561	Glycerolipid metabolism	0.74 (0.71–0.78)	1.58 (1.35–1.85)	0.07
00980	Metabolism of xenobiotics by cytochrome P450	0.69 (0.66–0.73)	1.2 (0.98–1.51)	0.07
04620	Toll-like receptor signaling pathway	0.74 (0.71–0.76)	1.56 (1.35–1.71)	0.07
04150	mTOR signaling pathway	0.72 (0.69–0.75)	1.43 (1.2–1.59)	0.08
00590	Arachidonic acid metabolism	0.7 (0.67–0.73)	1.27 (1.04–1.49)	0.1
00252	Alanine and aspartate metabolism	0.67 (0.64–0.7)	1.07 (0.85–1.27)	0.11
00532	Chondroitin sulfate biosynthesis	0.72 (0.69–0.76)	1.43 (1.21–1.74)	0.12

* See Table 2 for details on column headers.

KEGG, Kyoto Encyclopedia of Genes and Genomes (www.genome.jp/kegg).

### Balancing Model Complexity vs. Model Fit

It is important to note that a pathway with more SNPs will tend to have a higher nominal AUC than a pathway with less SNPs. This is a manifestation of the well-known relationship between model fit and model complexity [Gentle et al., 2004]; however, in this particular circumstance we cannot simply maximize, e.g., the Bayesian information criterion [Schwarz, 1978] because the number of predictors (namely SNPs) is fixed in a given pathway. Instead of doing this, we used two permutation controls. The first control was to permute the rows of the response variable *Y* and rerun the regression 500 times for each pathway matrix. This control consistently yielded low AUCs for every pathway, indicating that the pathway matrices were not large enough to spuriously fit a random response variable. The second and more rigorous control was a permutation-based approach to test whether the collection of SNPs in pathway *k* carried more predictive value than the same number of SNPs selected at random. To do this, for each pathway *k*, we first determined the number of statistically independent SNPs *jk* by using the Solid Spine algorithm in Haploview [Barrett et al., 2005] to assign SNPs to haplotype blocks in the pathway dosage matrix. The number of independent SNPs *jk* in pathway *k* was the number of haplotype blocks plus the number of SNPs not assigned to blocks. We then sampled *jk* SNPs from the pool of all SNPs with minimum P values below 0.05, which corresponded to permuting the SNP to pathway mapping. We concatenated the corresponding SNP columns to form a random dosage matrix with *jk* columns and computed the 10-fold bootstrap cross-validated median AUC as described above. We repeated this process 500 times to obtain the empirical conditional distribution *P*(*A*|*J*), where *A* was the AUC (the model fit) and *J* was the number of SNPs in the pathway (the model complexity). In practice, there were often multiple pathways with, e.g., 50 independent SNPs and no pathways with 53 independent SNPs; to compensate for this we ensured that exactly 500 median AUCs were calculated for each possible *J* value where *J* ranged from 1 to the maximum number of independent SNPs in all observed pathways.

### Smoothing Empirical Quantiles

The intent of doing this control was to compare the nominal AUC *a_k_* to the distribution of random AUCs for matrices of the same size, to determine a corrected P-value for pathway significance. Intuitively, a pathway containing, e.g., five independent SNPs that has a better nominal AUC than 495 out of 500 random matrices of five independent SNPs can be said to have an corrected P value of approximately 5/500=0.01, which is the empirical quantile [Koenker, 2007]. However, the straightforward approach of using empirical quantiles to compute corrected P values presents a minor dilemma. We expect that the threshold for a significant model fit should increase monotonically with the model complexity. But if we naively calculate the conditional empirical quantiles Q_α_[A|*J*] for each value of *J* = ∈ {1, … *j*_max_} and α = ∈ [0, 1], because of sampling variance we will with high probability encounter a situation in which the quantiles are no longer monotonically increasing:


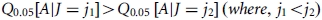
(3)

This corresponds to the undesirable situation in which the threshold for significance of the model fit at the α = 0.05 level is *decreasing* despite an increase in the number of independent SNPs in the pathway. To avoid this, we used quantile regression [Koenker, 2007] to fit a nondecreasing set of quantile curves (Fig. 2) to the *A* vs. *J* scatterplot. Superimposing the (*a_k_*, *jk*) values upon these curves gives us an immediate, visual diagnosis of which pathways have a model fit that is statistically significant relative to their number of independent SNPs (Fig. 2). These significance levels are enumerated in Tables 2 and 3 for both studies. Note that an alternative to this correction based on empirical quantiles of a permutation distribution would have been to use linear regression to partial out the effect of the number of independent SNPs on the nominal AUC, using the rank ordering of the resulting residuals as an ordering of pathway association strengths. However, correcting the nominal AUC-induced rankordering in this fashion would require the assumption of a linear relationship between the number of independent SNPs and the nominal AUC, an assumption which Figure 2 indicates is clearly violated.

## Results

We performed the SNP-level analyses for our dataset as well as the Fung et al. [2006] and Lesnick et al. [2007] datasets. Because individual-level data for the Lesnick et al. [2007] study was not available at the time of writing, we only conducted pathway-level analyses for our study and Fung et al.'s [2006] study.

### Replication of the Axon Guidance Pathway Association

As shown in Figure 2A and Table 2, one of our most interesting findings was that the axon guidance pathway (KEGG 04360) previously reported by Lesnick et al. [2007] as associated with PD came up as the third most significant pathway in our study after controlling for the number of SNPs, with ubiquitin proteolysis (KEGG 04120), histidine biosynthesis (KEGG 00340), and T-cell receptor signaling (KEGG 04660) at 1, 2, and 4, respectively. Though the apparent significance of ubiquitin proteolysis is intriguing given the known biology of PD [Rubinsztein, 2006], there were only two SNPs in this significant pathway (Table 4).

More puzzling, though, as shown in Figure 2B and Table 3, we find that axon guidance does not rank highly in the Fung et al. [2006] study after controlling for pathway size. Instead, the tight junction (KEGG 04530) and the pathogenic *E. coli* enterohemorrhagic *E. coli* (EHEC) and enteropathogenic *E. coli* (EPEC) pathways (KEGG 05130 and 05131, respectively) are the most highly ranked, despite an identical analytic approach. This lack of association between axon guidance and PD in the Fung et al. [2006] dataset may appear to contradict the results of Lesnick et al. [2007], but there is a key difference. In the Lesnick et al. [2007] study, the nominal axon guidance pathway association was computed from a set of SNPs thresholded for main effects with P values better than 0.05, but the significance of the association was tested by sampling from a pool of SNPs that was not thresholded for main effects.

Using our notation, this results in a downshifted permutation distribution of *P*(*A*|*J*) and hence an inflated empirical P value for the significance of any given pathway. While entirely understandable given that their work was a “candidate pathway” analysis rather than a comparative analysis of pathway significance, it nonetheless points to the necessity of systematically controlling for the number of SNPs in a pathway (model complexity) when evaluating the significance of a candidate pathway association (model fit).

### SNP Frequencies Globally Replicate

**Figure 3:**
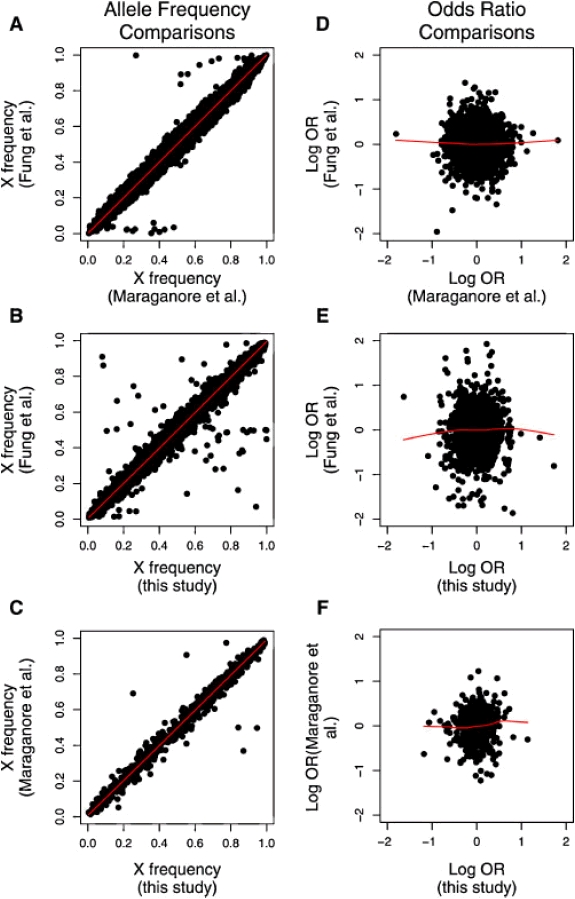
SNP allele frequencies correlate between studies but odds ratios do not. In all panels, points represent SNPs. **A–C:** Scatterplots of SNP allele frequencies between all three studies. **D–F:** Scatterplots of log odds ratios between studies. In each panel a locally linear LOWESS fit is superimposed. As noted in the text, allele frequencies clearly correlate at a global level but odds ratios do not.

Spurred by the finding that the axon guidance association appeared to partially but not completely replicate, we performed a detailed comparison of the three datasets, as summarized in Figure 3. We began by checking whether a disparity in assayed SNPs could account for the discrepancy. In Figure 3A–C, we see that SNP frequencies mostly match for the SNPs shared between our study and the Maraganore et al. [2005] and Fung et al. [2006] studies. This result is heartening because the underlying technologies are quite different: Fung et al. [2006] assayed 408,803 SNPs on the Illumina Infinium I and HumanHap300 platforms; Lesnick et al. [2007] assayed 248,535 SNPs on a Perlegen array; and we assayed 19,628 SNPs on the MegAllele Genotyping Human 20 K cSNP platform. Nevertheless, Figure 3A–C indicates technical replication of SNP frequencies between the studies.

Interestingly, we also observed that even SNPs that were out of HWE in one or more studies tend to replicate in frequency between studies. For example, the conditional correlation in allele frequencies between our study and Fung et al. [2006] for SNPs out of HWE is 0.919 (95% CI, 0.903–0.933), which is fairly high; in comparison, the conditional correlation in allele frequencies that do not deviate from HWE is 0.9927 (95% CI, 0.9923–0.9931). Similar tendencies hold for individual genotype frequencies. Thus, though deviation from HWE has been recommended as a quality control step in WGA [Leal, 2005], simply dropping SNPs that deviate from HWE may be premature. In particular, reproducible deviations from Mendelian inheritance patterns may indicate the presence of a copy number variant [Estivill and Armengol, 2007].

### SNP and Pathway Associations Do Not Globally Replicate

**Figure 4:**
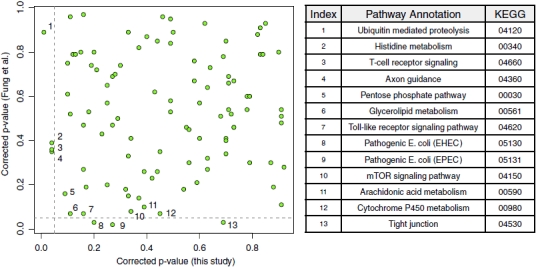
Pathway associations do not globally correlate between studies. In the plot, each point represents a pathway, and the vertical and horizontal dashed lines represent the P=0.05 thresholds in our study and the study by Fung et al. [2006], respectively. Pathways near the significance boundary are labeled and described in the right hand table. No pathway is significant in both our study and Fung et al.'s [2006] study (lower left corner), though glycerolipid metabolism (KEGG:00561) is closest.

In Figure 3D–F, we see that SNP associations do not globally correlate between studies. These results might be partially rationalized by noting that only a small set of SNPs might be expected to replicate, with all other associations at the level of noise; however, if this were the case we would expect to see a mixture distribution for the log odds ratio scatterplot, which consisted of a large bivariate normal of noisy, nonreplicated SNPs in the center and a small, tapering line of SNPs in the upper right and lower left corner of the plot, with reproducibly positive/negative log odds ratios. This tapering line is not observed. Figure 4 is a global comparison of pathway-level associations against the same disease between studies. Some have hypothesized that pathways replicate, but SNPs do not, by analogy to similar results in expression analysis [Subramanian et al., 2005]; however, in this case no overall correlation is apparent. Ubiquitin proteolysis, for example, is the most significant in our study and among the least significant in Fung et al. [2006]. It is, however, interesting to note that glycerolipid metabolism (KEGG 00561) is near the boundary of significance in both studies.

## Discussion

We have developed a method for ranking pathways by their degree of association with a trait of interest, which controls for the number of SNPs in the pathway. We applied this method to our own WGAS as well as two recent studies by Fung et al. [2006] and Maraganore et al. [2005]. We showed that pathway associations can be directly compared to SNP associations by calculating an equivalent odds ratio, and that in general the log odds ratios for pathway-level associations (Table 2) are higher than those for single SNPs; this is both expected and desirable, as pathway-level associations are based on several SNPs. Top-ranking pathways included ubiquitin proteolysis, histidine biosynthesis, T-cell receptor signaling, and axon guidance. In a parallel study, we performed a high-resolution linkage analysis, using a 500 K SNP chip, on familial Parkinsonian-pyramidal syndrome and could identify *FBXO7* (MIM# 605648) as the likely disease-causing gene [Shojaee et al., 2008]. Interestingly, *FBXO7* is part of the E3-ligase complex which is involved in ubiquitin-proteasome pathway, providing yet another validation of the importance of this pathway in the etiology of PD. We also found that the SNPs that are highly ranked in a univariate context (Table 1) are not the most significant SNPs in a multivariate, pathway context (Table 4). Both of these findings indicate that the simultaneous use of multiple SNPs is likely to increase diagnostic reproducibility over the use of one SNP at a time, a result similar to that found with expression microarrays [Subramanian et al., 2005]. As a particular example of a potentially reproduced result, we found partial replication of the previously reported axon guidance/PD association [Lesnick et al., 2007] in our study (Fig. 2), but observed that the overall reproducibility of SNP and pathway associations is poor (Figs. 3D–F and 4). Though it is tempting to state that differences in assayed SNPs are responsible for the discrepancies between pathway associations in our study and the Fung et al. [2006] study, Figure 3 partially argues against this conclusion as shared SNPs appear quite similar in terms of allele (and genotype) frequencies. It is more likely that the difference between the studies is in the genotype-to-phenotype map rather than the genotype matrices themselves; using the notation in Figure 1, this corresponds to a difference in *P*(*Y*|*X*) rather than in *P*(*X*).

The discrepancy between associated pathways can likely be remediated by modifying current protocols for genotyping, phenotyping, and data analysis. In terms of phenotyping, we recommend that future studies assay many more phenotypes, including molecular and biochemical traits (e.g., blood levodopa levels), in addition to high-level Boolean phenotypes such as case/control status. This is because basic phenotypes are easier to explain in terms of molecular variation; for example, it is easier to predict expression levels [Spielman et al., 2007] from SNP data than it is to predict height [Visscher et al., 2007]. With respect to genotyping, we believe that future studies should involve as many different features as possible in addition to SNP genotyping, including expression arrays and copy number variants [Estivill and Armengol, 2007]. In particular, we expect that studies of standing variation in copy numbers will produce more consistent matches to existing molecular results because manipulation of gene dosage (via knockout, knockdown, and overexpression) is the stock-in-trade of molecular biology.

Finally, with respect to data analysis, the discrepancies observed here between different studies are reminiscent of the early days of microarrays. There, too, conflicting results [Miklos and Maleszka, 2004] were eventually resolved after many large data sets were combined with the adoption of a systematic, Bayesian integration strategy [Jansen et al., 2003; Lee et al., 2004; Srinivasan et al., 2007]. A similar integration methodology in the context of WGA is a fruitful topic for further research.
